# Functional validation of novel *MKS3/TMEM67* mutations in COACH syndrome

**DOI:** 10.1038/s41598-017-10652-z

**Published:** 2017-08-31

**Authors:** So-Hyun Lee, Tai-Seung Nam, Wenting Li, Jung Ha Kim, Woong Yoon, Yoo-Duk Choi, Kun-Hee Kim, Hua Cai, Min Jung Kim, Changsoo Kim, Hyon E. Choy, Nacksung Kim, Kee Oh Chay, Myeong-Kyu Kim, Seok-Yong Choi

**Affiliations:** 1 Department of Biomedical Sciences, Chonnam National University Medical School, Gwangju, Republic of Korea; 2 Center for Creative Biomedical Scientists at Chonnam National University, Gwangju, Republic of Korea; 3 Department of Neurology, Chonnam National University Medical School, Gwangju, Republic of Korea; 4 Department of Pharmacology, Chonnam National University Medical School, Gwangju, Republic of Korea; 5 Department of Radiology, Chonnam National University Medical School, Gwangju, Republic of Korea; 6 Department of Pathology, Chonnam National University Medical School, Gwangju, Republic of Korea; 7 Department of Microbiology, Chonnam National University Medical School, Gwangju, Republic of Korea; 8 Department of Biological Sciences, Sookmyung Women’s University, Seoul, Republic of Korea; 9 School of Biological Sciences and Technology, Chonnam National University, Gwangju, Republic of Korea; 10 Department of Biochemistry, Chonnam National University Medical School, Gwangju, Republic of Korea

## Abstract

COACH syndrome is an autosomal recessive developmental disorder, a subtype of Joubert syndrome and related disorders, characterized by cerebellar vermis hypoplasia, oligophrenia, ataxia, coloboma, and hepatic fibrosis. Although mutations in *TMEM67 (transmembrane protein 67)/MKS3 (Meckel-Gruber syndrome, type 3)* were reported to cause COACH syndrome, this causality has not verified by functional studies. In a 20-year-old Korean man, we found cerebellar ataxia, isolated elevation in serum γ-glutamyl transpeptidase (γ-GTP) activity, oligophrenia, the molar tooth sign (MTS) in the brain MR images and congenital hepatic fibrosis (CHF). Two novel compound heterozygous mutations were found in *TMEM67* in the patient: i) missense mutation (c.395 G > C and p.Gly132Ala) in exon 3, and ii) deletion in exon 26 (c.2758delT and p.Tyr920ThrfsX40). Western blotting showed that the p.Tyr920ThrfsX40 mutation accelerates turnover of the TMEM67 protein. Although wild-type human *TMEM67* RNA rescued phenotypes of zebrafish embryos injected with anti-sense oligonucleotide morpholinos against *tmem67*, the two human *TMEM67* RNAs individually harboring the two mutations did not. Finally, Wnt signaling, but not Hedgehog signaling, was suppressed in *tmem67* morphants. To the best of our knowledge, this is the first report verifying the causality between COACH syndrome and *TMEM67*, which will further our understanding of molecular pathogenesis of the syndrome.

## Introduction

Joubert syndrome (JBTS), first described in 1969 by Joubert and colleagues^1^, is a rare neurodevelopmental disorder with a complex mid-hindbrain malformation characterized by the “molar tooth sign” (MTS) on axial brain magnetic resonance (MR) images^2, 3^. The MTS consists of i) a deepened interpeduncular fossa, ii) thick, elongated and mal-oriented superior cerebellar peduncles, iii) a fourth ventricle anomaly, and iv) a dysplastic cerebellar vermis^4, 5^. Typically, patients with classic JBTS manifest the cardinal neurological features of ataxia, hypotonia, abnormal ocular movements, oligophrenia (developmental delay/mental retardation), and irregular breathing/central sleep apnea^3, 6^. “JBTS and related disorders (JSRDs)” are clinically heterogeneous conditions sharing the MTS with multiple organ involvement, such as retina, kidneys, liver, and skeleton. One type of JSRD with neurological signs of JBTS and congenital hepatic fibrosis (CHF) is called the COACH syndrome (Cerebellar vermis hypoplasia, Oligophrenia, Ataxia, Coloboma, and Hepatic fibrosis; Online Mendelian Inheritance in Man [OMIM] # 216360)^3, 5, 6^. Of note, chorioretinal or optic nerve coloboma does not appear to be an essential feature of COACH syndrome, as Doherty and colleagues have reported COACH syndrome patients without coloboma^7^.

**Figure 1:**
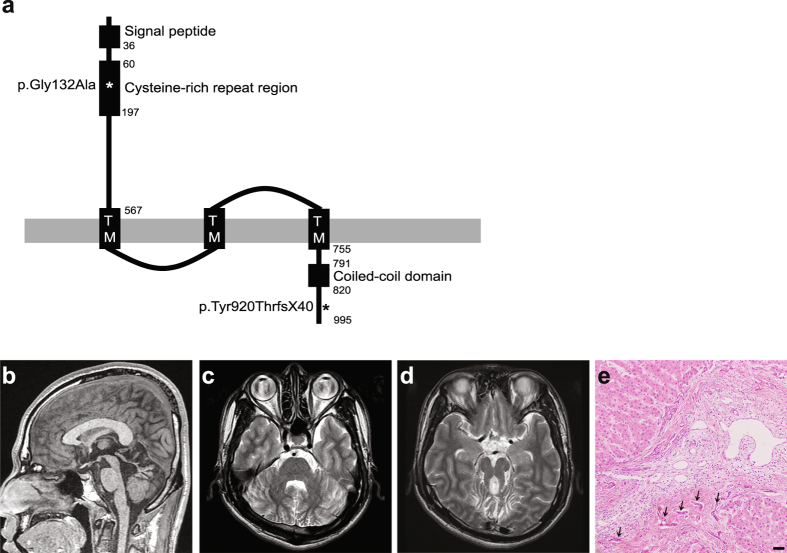
Domain structure of TMEM67 and clinical images of the patient with COACH syndrome. (**a**) Domain structure of TMEM67. Reference TMEM67 sequence is NCBI accession number NP_714915.3. Numbering indicates the amino-acid residue. TM: transmembrane domain. Asterisks represent the two mutations identified in this study. Not drawn to scale. (**b**) T1-weighted sagittal MR image shows a very small dysplastic vermis located superiorly. (**c**) T2-weighted axial MR image shows a midline vermian clefting. (**d**) T2-weighted axial MR image at the level of pons-midbrain junction shows the characteristic appearance of the molar tooth sign with a deep interpeduncular fossa, elongated thick and mal-oriented superior cerebellar peduncles, and dysplastic cerebellar vermis. (**e**) The liver biopsy specimen stained with hematoxylin and eosin exhibits hepatic fibrosis, bile ductule proliferation (arrows) and vascular dilatation. Scale bar = 5 μm.

The clinical manifestations of CHF in COACH syndrome may vary from an asymptomatic increase in serum hepatic enzymes or early onset hepatosplenomegaly to more severe manifestations including portal hypertension, liver cirrhosis and esophageal varices^8^. To date, it has been reported that COACH syndrome is primarily caused by mutations in the *Transmembrane Protein 67* gene (*TMEM67*; also called *Meckel-Gruber syndrome type 3* gene [*MKS3*] or *meckelin*) and rarely by mutations in the *RPGRIP1L* (also called *NPHP*) or *CC2D2A* gene^7, 9^. TMEM67 is mostly localized to the primary cilia, especially the ciliary membranes that covers the axoneme of the primary cilia^10^. Human TMEM67 has 995 amino acid residues and harbors four kinds of domains: a signal peptide, a cysteine-rich repeat region, three transmembrane domains and a coiled-coil domain, from N- to C-terminus (Fig. 1a)^11, 12^. The extracellular cysteine-rich repeat region is rich in cysteine residues, akin to the cysteine-rich domain of Frizzled^10^, which is implicated in canonical and non-canonical Wnt signaling. Of note, disruption of Wnt signaling is frequently observed with ciliopathies^13, 14^. The coiled-coil domain seems to be involved in interaction with other proteins, such as Nesprin-2 (nuclear envelope spectrin repeat protein 2)^11^. Mutations were identified throughout the *TMEM67* gene in COACH syndrome patients^7^. Mice lacking *Tmem67* show polycystic kidney disease, hydrocephalus, pulmonary hypoplasia, ventricular septal defects, limb dysplasia and brain anomaly including cerebellar hypoplasia^15–17^.

Here, we report two novel mutations (p.Gly132Ala and p.Tyr920ThrfsX40) in *TMEM67* in a Korean patient with clinical manifestations of JBTS and CHF.

## Methods

### Patients

This study was exempt from review by the Institutional Review Board at Chonnam National University Hospital (CNUH-2012-232) as it involved the research of existing data. In addition, this study was conducted in accordance with the 1964 Declaration of Helsinki and its later amendments. Written informed consents for the use of the medical records and *TMEM67* gene sequences were obtained from all the participants including members of the proband’s family.

### Reagents

All chemicals were purchased from Sigma-Aldrich (St. Louis, MO, USA), unless indicated otherwise.

### DNA analysis

Genomic DNA was extracted from peripheral blood leukocytes of the patient by phenol-chloroform extraction^18^. The entire 28 coding exons and the exon-intron boundaries of the *TMEM67* were amplified by polymerase chain reaction (PCR) (Supplementary Table 1). The resulting PCR products were directly sequenced as described previously^19^. Multiple amino acid alignment was performed by ‘COBALT Clustal (http://www.ncbi.nlm.nih.gov/tools/cobalt/re_cobalt.cgi).’ PolyPhen-2 (http://genetics.bwh.harvard.edu/pph2/) was used to predict the functional consequences of amino acid substitutions in TMEM67. The identified TMEM67 mutations, p.Gly132Ala and p.Tyr920ThrfsX40, were submitted to the ‘Leiden Open Variation Database (http://www.lovd.nl).’

### DNA manipulation

An expression plasmid encoding human TMEM67 fused C-terminally to a FLAG epitope (p3xFLAG-CMV-14-human TMEM67) was kindly provided by Timothy Weaver^20^. The reference sequence for *TMEM67* on this plasmid was based on the GenBank sequence BC032835.2 (protein: AAH32835.1 [985 amino acid residues]). Of note, BC032835.2 lacks 30 nucleotides encoding the N-terminal 10 amino acids (residues 1–10) of the *TMEM67* reference sequence NM_153704.5 (protein: NP_714915.3 [995 amino acid residues]), which was the reference used in this study.

Plasmids encoding TMEM67(p.Gly132Ala) and TMEM67(p.Tyr920ThrfsX40) were generated by site-directed mutagenesis. Primers used are listed in Supplementary Table 2. Wild-type (WT) and mutant alleles of *TMEM67* were individually subcloned into the EcoRV and BglII sites of the pCS4 + vector^21^. All plasmids constructed were verified by DNA sequencing (Macrogen, Daejeon, Korea).

### Histopathology

Liver tissue was obtained by ultrasound-guided liver biopsy of the patient, embedded in paraffin, serially sectioned at 3-µm thickness, and then stained with hematoxylin and eosin (H&E).

### Cell culture and Western blotting (WB)

HEK293T cells were purchased from American Type Culture Collection (Manassas, VA, USA) and cultured in Dulbecco’s Modified Eagle’s Medium (DMEM; Welgene, Korea) supplemented with 10% fetal bovine serum (FBS; GIBCO, Carlsbad, CA, USA). The cells were plated in 6-well plates at a density of 2 × 10^5^ cells/well 24 hr prior to transfection. Plasmids encoding WT or mutant TMEM67 were transfected into the cells using FuGENE 6 (Promega, Madison, WI) as per the manufacturer’s instructions. At 48 hr post-transfection, the cells were washed with phosphate buffered saline (PBS) and then lysed with extraction buffer (50 mM Tris-HCl [pH 8.0], 150 mM NaCl, 1 mM EDTA, 0.5% Nonidet P-40, 1 mM PMSF, and protease inhibitor cocktail). Equal amounts of proteins were subjected to sodium dodecyl sulfate polyacrylamide gel electrophoresis (SDS-PAGE) and then transferred to a PVDF membrane (Millipore, Billerica, MA, USA). The membrane was probed with the indicated antibody conjugated to horseradish peroxidase (HRP) (anti-FLAG [1:5000, Sigma, catalog number A8592] and anti-β-actin [1:20,000, Sigma, catalog number A3854]), and then washed with TBST (10 mM Tris [pH 7.6], 150 mM NaCl and 0.1% Tween-20). Finally, the bound antibody was detected by enhanced chemiluminescence (Millipore) and analyzed with a LAS3000 luminescent image analyzer (GE Healthcare, Piscataway, NJ, USA). For a protein stability assay, the HEK293T cells transfected as described above were treated with MG132 (25 μM) for 4 hr before harvest.

### Zebrafish study

All animal experiments were approved by the Chonnam National University Medical School Institutional Animal Care and Use Committee (IACUC), and conducted in accordance with relevant guidelines and regulations in Republic of Korea. Wild type zebrafish (AB strain) were obtained from the Zebrafish International Resource Center (Eugene, OR), maintained using standard procedures^22^ and staged in days post-fertilization (dpf) as per standard criteria^23^. Morpholino oligonucleotides (MOs) targeting the exon 2-intron 2 junction [e2i2] of *TMEM67* (5′-ATC CAA AAT AAA TAC AGC ACC TGA T-3′) and MOs targeting the exon 3-intron 3 junction [e3i3] of *TMEM67* (5′-GTA AAA ATG ACA AGC GCC TAC CCA G-3′) were purchased from Gene Tools (Corvallis, OR, USA). Control MOs (5′-CCT CTT ACC TCA GTT ACA ATT TAT A-3′) were also purchased from Gene Tools. The MO (4 ng) was injected into WT or *Tg(Tcf/Lef-miniP:dGFP)* ^*isi01*^ ^24^ one-cell stage embryos, incubated in egg water until the indicated stage and then processed for further experiments.

To test the efficiency of *tmem67* MO, embryos at 1 dpf were anesthetized in 0.02% tricane, and total RNA was isolated using TRIzol (Molecular Research Center, Cincinnati, OH, USA) from morphants. First strand cDNA was then synthesized from the resulting RNA using SuperScript III First-Strand Synthesis System for RT-PCR (Thermo Fisher Scientific Korea, Seoul, Korea) and the cDNA fragment encompassing exons 1–5 or 2–6 was PCR-amplified with a pair of primers (Supplementary Table 3) as described previously^25^.

For the rescue experiment, one-cell stage embryos were sequentially injected with the MO (4 ng) followed by RNAs (50 pg) synthesized from plasmids individually encoding the WT or indicated TMEM67 mutant allele as described previously^26^. Finally, the embryos were imaged with a SteREO Discovery V20 stereomicroscope (Carl Zeiss, Jena, Germany).

Whole-mount *in situ* hybridization (WISH) was performed with *patched (ptch) 1*, *ptch2, gli1 and axin2* riboprobes as described previously^27^. Plasmids encoding zebrafish *ptch1*, *ptch2 and axin2* were provided by Hyunju Ro, and *gli1* by Rolf O. Karlstrom^28^.

### Quantitative real-time PCR (qPCR)

Total RNAs were extracted from transfected HEK293T cells or injected zebrafish embryos at 2.5 dpf (60 embryos / group) using TRIzol. First strand cDNA was then synthesized from the resulting RNA using a TOPscript cDNA Synthesis Kit (enzynomics, Daejeon, Korea), added to TOPreal qPCR 2x PreMIX (SYBR Green with high ROX; enzynomics) and indicated primers (Supplementary Table 4), and analyzed using a Rotor-Gene Q Real-time PCR System (Qiagen; Valencia, CA, USA). Expression levels of indicated mRNA were normalized to those of actin primers.

### Image analysis

The images taken were assembled using Adobe Photoshop (San Jose, CA, USA) and the size of the hindbrain ventricle of zebrafish embryos was measured using ImageJ (National Institutes of Health, Bethesda, MD, USA).

### Statistical analysis

The *p* value^29^ was determined by the two-tailed unpaired Student′s t-test.

## Results

### Clinical features

The patient was a 20-year-old male college student who had been treated from his late teens with ursodeoxycholic acid due to an increase in serum γ-GTP activity of unknown etiology. He had a history of delayed developmental milestones: he was able to speak and walk upright at 5 and 7 years of age, respectively. He had difficulty running due to unsteadiness on his feet from his teens. There was no family history of gait disturbances, psychomotor retardation or consanguinity.

On admission, icteric sclera, jaundice, hepatomegaly and ascites were absent. His neurological examination revealed scanning dysarthria, see-saw nystagmus, positive finger-to-nose test and inability of tandem gait. Ophthalmological examination did not reveal retinal dystrophy, chorioretinal or optic nerve coloboma. In addition, video-oculography showed oculomotor apraxia, pendular see-saw nystagmus, gaze-evoked nystagmus and saccadic hypometria. The liver function test showed that alanine aminotransferase (ALT) was 45 U/L (normal range < 42 U/L); aspartate aminotransferase (AST), 36 U/L (normal range < 38 U/L); alkaline phosphatase, 236 U/L (normal range < 129 U/L); and γ-GTP, 763 U/L (normal range < 61 U/L). His serum bilirubin, prothrombin time, albumin, blood urea nitrogen and creatinine were within normal limits. No serological evidence of viral or autoimmune hepatitis was noted. To evaluate oligophrenia, Korean Mini-Mental Status Examination (K-MMSE) was performed and the score was 29, which is within normal limits. In addition, intelligence quotient (IQ) was assessed by Korean Wechsler Adult Intelligence Scale (K-WAIS), a Korean edition of the revised form of WAIS: the patient scored 51 in full scale IQ (verbal IQ: 57 and performance IQ: 53), indicating mild to moderate mental retardation.

Computed tomography of the abdomen showed multiple nephrolithiasis in both kidneys without evidence of hepatosplenomegaly, liver cirrhosis or portal hypertension. Brain MR images demonstrated MTS consisting of dysplastic cerebellum, vertically oriented superior cerebellar peduncles, deep interpeduncular fossa and widening of the fourth ventricle (Fig. 1b–d). Ultrasound-guided liver biopsy exhibited a hepatic parenchymal fibrosis, bile ductule proliferation, and vascular dilatation, suggestive of CHF (Fig. 1e).

Based on the MTS in the brain MR images, oligophrenia and CHF, albeit no coloboma, the patient was diagnosed with COACH syndrome.

### DNA analysis of *TMEM67*

**Figure 2:**
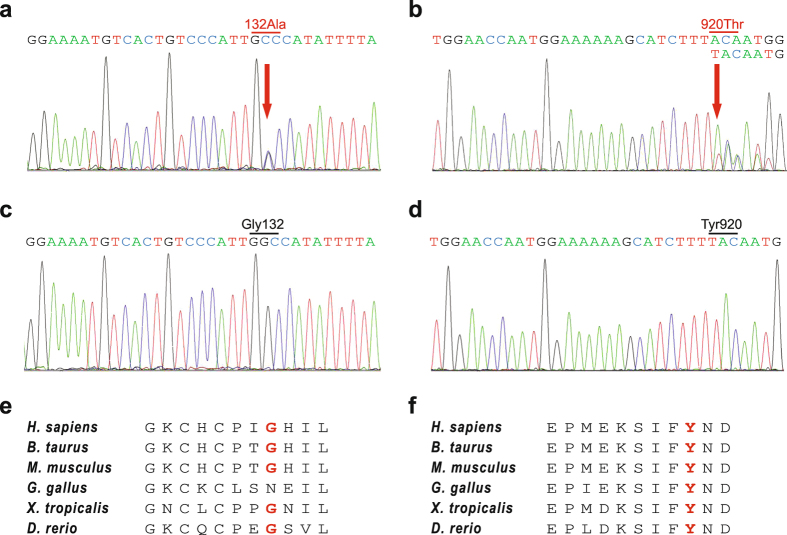
DNA sequence analysis of *TMEM67* gene and amino acid alignment of evolutionarily distant TMEM67 orthologs. (**a** and **b**) Electropherograms of *TMEM67* with novel mutations at positions 395 bp (p.Gly132Ala) and 2758 bp (p.Tyr920ThrfsX40). (**c** and **d**) Representative electropherograms of *TMEM67* from 200 normal controls. (**e** and **f**) Conservation of Gly132 and Tyr920 residues (red) from zebrafish to humans. The alignment was performed using Cobalt Constraint-based Multiple Protein Alignment Tool (http://www.ncbi.nlm.nih.gov/tools/cobalt/re_cobalt.cgi). GenBank accession numbers of the orthologs are as follows: *Homo sapiens* (NP_714915.3), *Bos taurus* (cattle; NP_001192228.1), *Mus musculus* (house mouse; NP_808529.2), *Gallus gallus* (chicken; XP_418334.3), *Xenopus tropicalis* (tropical clawed frog; XP_002934466.2) and *Danio rerio* (zebrafish; XP_700974.4).

Under the provisional diagnosis of COACH syndrome, the patient’s *TMEM67* coding region was sequenced and compound heterozygous mutations were found: i) a heterozygous substitution G to C in exon 3 at 395 bp (c.395G > C), where 1 bp corresponded to the first nucleotide of the ATG start codon in the cDNA reference sequence (NCBI accession number NM_153704.5), causing an amino acid change at codon 132 (p.Gly132Ala; protein coordinates: NCBI NP_714915.3) (Fig. 2a and c) and ii) a heterozygous deletion in exon 26 at 2758 bp (c.2758delT), creating a frameshift that introduced a premature termination codon (PTC) at the 960th amino acid (p.Tyr920ThrfsX40) (Fig. 2b and d), thereby truncating the C-terminal 36 amino acids of TMEM67. Of note, p.Gly132Ala lies in the cysteine-rich region of TMEM67. The patient’s asymptomatic father had heterozygous p.Gly132Ala, but not p.Tyr920ThrfsX40. The patient’s unaffected mother and sister did not agree to undergo the genetic analysis. These two mutations occurred in amino acid residues that are highly conserved from human to zebrafish except for p.Gly132 in *Gallus gallus* (chicken) (Fig. 2e and f), and are predicted by PolyPhen-2 to be possibly (p.Gly132Ala) and probably damaging (p.Tyr920ThrfsX40). These two mutations were not found in the *TMEM67* of 200 normal South Koreans (Fig. 2c and d). Notably, p.Tyr920ThrfsX40 is listed as a single nucleotide polymorphism with unknown minor allele frequency (dbSNP ID number: rs777993921). Taken together, these findings suggest that the two mutations might alter the function of TMEM67.

### Functional studies of mutant *TMEM67* proteins

**Figure 3:**
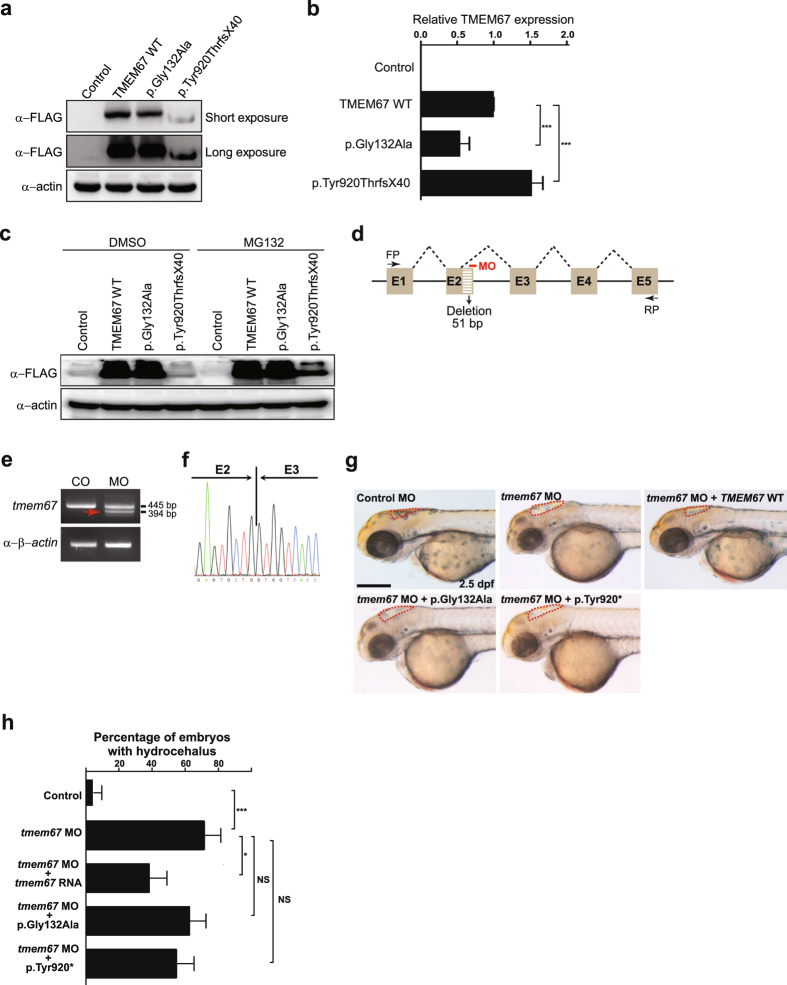
TMEM67 mutants show decrease in protein function. **(a)** HEK293T cells were transfected with plasmids individually encoding WT, p.Gly132Ala or p.Tyr920ThrfsX40 TMEM67, all fused C-terminally to a FLAG epitope, and processed for WB with anti-FLAG antibody. Anti-actin antibody was used for a loading control. Shown is a representative image of three independent experiments. Full-length blots are presented in Supplementary Fig. S1. **(b)** The transfected cells in **(a)** were processed for quantitative real-time PCR with *TMEM67*-specific primers. Expression levels of *TMEM67* were normalized to those of *ACTIN*. ***P < 0.001 by the two-tailed Student’s *t*-test (n = 3). **(c)** The HEK293T cells transfected as described in **(a)** were treated with DMSO (dimethyl sulfoxide; vehicle control) or MG132 (25 μM) for 4 hr before harvest and processed for WB as described in **(a)**. Cropped blots are presented. **(d)** A schema showing the binding site of *tmem67* splice-blocking morpholino (MO [e2i2]). Dashed lines indicate splicing events. Not drawn to scale. E: exon; FP: forward primer; RP: reverse primer. **(e)** One-cell stage zebrafish embryos were injected with *tmem67* MOs and processed for RT-PCR. β-actin was used as a loading control. Arrow indicates a PCR product with deletion of a part of exon2. **(f)** An electropherogram of an exon junction area of the PCR product indicated by arrow in **(e)**. **(g)** One-cell stage zebrafish embryos were injected with either control MOs or *tmem67* MOs [e2i2] and imaged at 2.5 days post-fertilization (dpf). For a rescue experiment, one-cell stage zebrafish embryos were sequentially injected with *tmem67* MO [e2i2] and RNA encoding the indicated TMEM67 mutant and imaged at 2.5 dpf. Dashed lines mark the hindbrain ventricles. Arrows indicate enlarged ventricles (hydrocephalus). p.Tyr920* represents p.Tyr920ThrfsX40. Scale bar = 100 μm. **(h)** Embryos with hydrocephalus were counted in each group in **(g)**. The hindbrain ventricles larger than 5,000 μm^2^ were considered to be hydrocephalic. *P < 0.05; ***P < 0.001 by the two-tailed Student’s *t*-test; NS: not significant. Number of larvae used in the analysis of each group is over 40.

Given the recessive nature of COACH syndrome, it stands to reason that *TMEM67* mutations eliciting COACH syndrome are loss-of-function mutations. As reduced protein stability is one of the most common causes of loss of protein function, we sought to assess the stability of mutant TMEM67 proteins. To this end, we first transfected HEK293T cells with expression plasmids individually encoding WT, p.Gly132Ala, or p.Tyr920ThrfsX40 TMEM67, all of which were C-terminally fused to a FLAG epitope, and processed the cells for WB with anti-FLAG antibody. While expression levels of p.Gly132Ala were slightly decreased compared to those of WT, expression levels of p.Tyr920ThrfsX40 were markedly reduced compared to those of WT (Fig. 3a and Supplementary Fig. S1). To determine whether decrease in p.Gly132Ala and p.Tyr920ThrfsX40 stems from reduced RNA or protein levels, we first assessed using qPCR RNA levels of WT, p.Gly132Ala and p.Tyr920ThrfsX40 *TMEM67* in HEK293T cells transfected with respective plasmids. Levels of p.G132A RNA were significantly diminished compared to those of WT *TMEM67* RNA, indicating that slight reduction in p.Gly132Ala may ensue from RNA instability. However, levels of p.Tyr920ThrfsX40 RNA were significantly enhanced compared to those of WT *TMEM67* RNA (Fig. 3b), which might be caused by a compensatory mechanism. We then tested if treatment of HEK293T cells with a proteasome inhibitor, MG132, could blunt the decrease in p.Tyr920ThrfsX40 levels. MG132 treatment partially restored p.Tyr920ThrfsX40 levels (Fig. 3c), suggesting that the diminution in p.Tyr920ThrfsX40 levels may be caused by protein instability, at least in part.

To further test if p.Gly132Ala and p.Tyr920ThrfsX40 are pathogenic, we turned to a zebrafish model where MO knockdown of *tmem67* leads to various morphological defects characteristic of ciliopathy, such as hydrocephalus, pronephric cysts, curved trunk and notochord defect^30, 31^. Of these morphant phenotypes, we focused on hydrocephalus as it is the most noticeable one. We first designed MOs that bind to the junction between exon 2 and intron 2 (MOs [e2i2]) (Fig. 3d). Injection of these MOs into one-cell stage embryos altered the normal splicing pattern, as revealed by RT-PCR (Fig. 3e). DNA sequence analysis of the aberrant splicing product indicated that the altered splicing led to the activation of a cryptic splice site in the exon 2, thereby deleting 51 nucleotides (17 amino acids) (Fig. 3d and f), which validated the efficacy of the *tmem67* MOs. As reported previously^30, 31^, the *tmem67* MO induced hydrocephalus in zebrafish embryos. In addition, sequential injection of embryos with the *tmem67* MO followed by human WT *TMEM67* RNA significantly reduced number of embryos with hydrocephalus, confirming specificity of the *tmem67* MO [e2i2]. However, neither p.Gly132Ala nor p.Tyr920ThrfsX40 rescued the hydrocephalus phenotype (Fig. 3g and h). Furthermore, another MO targeting the junction between exon 3 and intron 3 (MOs [e3i3]) rendered comparable results to those from MOs [e2i2] (Supplementary Fig. S2), confirming the specificity of *tmem67* morphant phenotypes. Taken together with the WB and qPCR results in Fig. 3a–c, this outcome suggests that p.Gly132Ala and p.Tyr920ThrfsX40 may be pathogenic by diminishing the levels of TMEM67 and/or suppressing the function of TMEM67.

**Figure 4:**
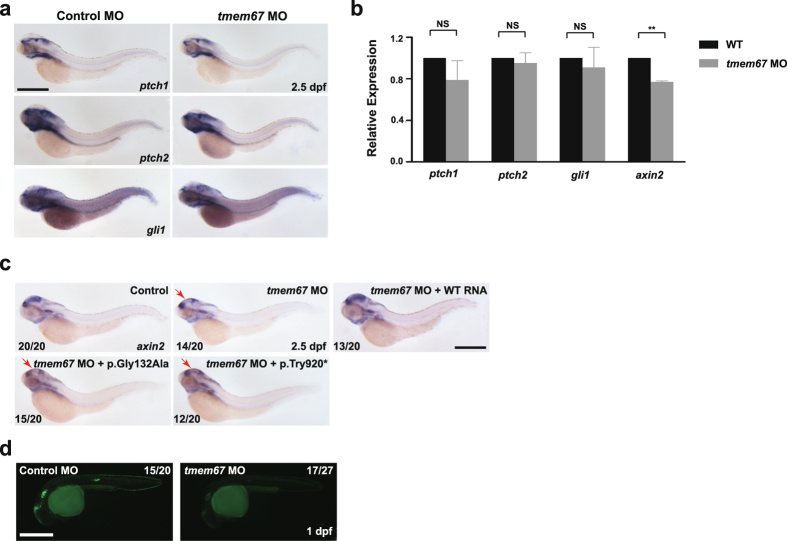
Wnt signaling, but not Hh signaling, is reduced in *tmem67* morphants. **(a)** Zebrafish embryos were injected with either control or *tmem67* MOs [e2i2], subjected to WISH at 2.5 dpf with *ptch1*, *ptch2* and *gli1* riboprobes, and imaged under a light microscope. NS: not significant. Scale bar = 200 μm. (**b**) Total RNAs were extracted from WT or *tmem67* morphant embryos at 2.5 dpf and subjected to quantitative real-time PCR with *ptch1*, *ptch2, gli1* and *axin2 primers*. Expression levels of indicated mRNA were normalized to those of actin primers. NS: not significant. ^**^ *P* < 0.01. **(c)** One-cell stage zebrafish embryos were injected with either control or *tmem67* MOs [e2i2], probed with *axin2* riboprobes at 2.5 dpf and imaged. For a rescue experiment, one-cell stage zebrafish embryos were sequentially injected with *tmem67* MO [e2i2] and RNA encoding the indicated TMEM67 mutant, probed with *axin2* riboprobes at 2.5 dpf and imaged. p.Tyr920* represents p.Tyr920ThrfsX40. Arrows indicate reduction of signals compared to those in control embryos. Scale bar = 200 μm. (**d**) *Tg(Tcf/Lef-miniP:dGFP)* embryos were injected with either control or *tmem67* MOs [e3i3] and imaged at 1 dpf under a fluorescent microscope. Scale bar = 200 μm.

As TMEM67 is mostly localized to the primary cilia^10^, and primary cilia are required for Hedgehog (Hh) and Wnt signaling^13, 14, 32^, we sought to assess Hh and Wnt signaling in *tmem67* morphants. We first performed WISH on 2.5-dpf embryos injected with *tmem67* MO [e2i2] with *ptch1*, *ptch2* and *gli1* riboprobes as they are target genes of Hh signaling^28, 33, 34^. No noticeable difference was noted in *ptch1*, *ptch2* and *gli1* expression between control embryos and *tmem67* morphants (Fig. 4a and b). However, WISH and qPCR revealed suppression of mRNA levels of *axin2*, a target gene of Wnt signaling^35^, in *tmem67* morphants [e2i2] compared to control embryos (Fig. 4b and c). Likewise, injection of *tmem67* MOs [e3i3] into Wnt reporter fish, *Tg(Tcf/Lef-miniP:dGFP)*, where GFP (green fluorescent protein) expression is a readout for Wnt signaling^24^, significantly reduced GFP expression in *tmem67* morphants compared to control embryos (Fig. 4d). Collectively, these findings indicate that TMEM67 may be implicated in Wnt signaling, but not in Hh signaling.

To ascertain if p.Gly132Ala and p.Tyr920ThrfsX40 can rescue diminished Wnt signaling, we sequentially injected into embryos MOs [e2i2] followed by RNAs encoding WT or mutant alleles of TMEM67 and carried out WISH on larvae at 2.5 dpf with *axin2* riboprobes. Although WT *TMEM67* RNA restored *axin2* mRNA levels to those in control embryos, neither p.Gly132Ala nor p.Tyr920ThrfsX40 did (Fig. 4c). This outcome implies that both p.Gly132Ala and p.Tyr920ThrfsX40 might alter Wnt signaling.

## Discussion

Here, we identified two novel compound heterozygous mutations (p.Gly132Ala and p.Tyr920ThrfsX40) in the *TMEM67* gene in a 20-year-old male patient with COACH syndrome. To functionally validate the two mutations, we co-injected into zebrafish embryos *tmem67* MO and RNAs encoding p.Gly132Ala and p.Tyr920ThrfsX40, and observed no rescue of the hydrocephalus phenotype and the decrease in *axin2*. These findings indicate that the two mutations may cause COACH syndrome via altered Wnt signaling, at least in part. Although most patients with COACH syndrome showed elevated liver enzymes and coloboma^7, 9^, the proband in the present study did not.

Primary (non-motile) cilia serve as sensory organelles. Despite their presence in almost all vertebrate cells, phenotypes of primary cilia dysfunction appear primarily in the kidney, brain, retina, bone and liver, the reason for which is still elusive^36–39^. In ciliopathies, mutations in the same gene can bring about different phenotypes^40, 41^. For example, mutations of *TMEM67* cause various genetic diseases such as Meckel-Gruber syndrome type 3 (OMIM # 607361), JBTS type 6 (OMIM # 610688), nephronophthisis type 11 (NPHP11; OMIM # 613550) and COACH syndrome^42^. What is the molecular mechanism by which mutations in the same gene cause various genetic diseases? Physical location of the mutations in the TMEM67 protein may not account for the difference in phenotypes based on the following reasons. First, Gleeson, Valente and colleagues reported a COACH syndrome patient (COR71) who had p.Pro130Arg and p.Trp225* compound heterozygous mutations in TMEM67. The Pro130 residue is very close to the G132 residue where we identified a mutation, and both of them lie in the cysteine rich region. However, COR71 had coloboma and nephronophthisis^9^, which our patient did not show. Second, Doherty and colleagues reported in COACH syndrome patients (UW52-3 and UW52-4) a mutation (p.Gly934GlyfsX26) in the G934 residue that is located in the C-terminal region of TMEM67, where the mutation we identified, p.Tyr920ThrfsX40, also resides. The COACH syndrome patient with p.Tyr920ThrfsX40 had no coloboma, whereas both UW52-3 and UW-52-4 had coloboma^7^. Intriguingly, in UW52-3 and UW52-4, p.Gly934GlyfsX26 was the only mutation found in TMEM67. These differences in phenotypes might ensue from genetic (or mutational) pleiotropy^43–46^ of TMEM67, the combination of mutations in TMEM67, genetic modifiers in other genes, or a subset, or all of the above. Further understanding of the molecular function of TMEM67 would help to uncover the mechanism by which mutations in TMEM67 cause various genetic diseases.

Primary cilia have been tied to Hh and Wnt signaling, yet there is controversy over the connection between cilia and Wnt signaling^37, 39, 47^. In the present study, we show that MO knockdown of *tmem67* reduces Wnt signaling, but not Hh signaling, in zebrafish embryos. Johnson and colleagues reported two phenotype groups in mouse embryos lacking *Tmem67*. An MKS-like group showed exencephaly, frontal/occipital encephalocele, increased Wnt signaling and decreased Hh signaling. A JBTS-like group manifested cerebellar hypoplasia, suppressed Wnt signaling and enhanced Hh signaling. They attributed this phenotype difference in *Tmem67* null mouse embryos (‘inter-individual variation’), at least in part, to genetic modifiers and stochastic effects^17^. Jagger and colleagues observed multi-organ developmental defects including pulmonary hypoplasia in *Tmem67* knockout mouse embryos that were reminiscent of those shown in *Wnt5a* knockout mouse embryos, proposing that TMEM67 curbs canonical Wnt and Hh signalings^16^. Harris and colleagues demonstrated that canonical Wnt signaling is upregulated in kidneys of *Tmem67* null mice^31^. Further study is warranted to determine the reason for this discrepancy in Wnt and Hh signalings among *Tmem67* knockout mice or between *Tmem67* knockout mouse embryos and *tmem67* morphant zebrafish embryos.

As mutations in TMEM67 are associated with ciliopathy phenotypes in JSRDs^16^, exploration of the structure or function of the cilia in COACH syndrome is warranted. Our report of two novel mutations in TMEM67 will widen the genetic spectrum of COACH syndrome, thereby contributing to understanding the molecular pathogenesis of COACH syndrome.

## Electronic supplementary material

Supplementary data
