# Supplementary material for: Functional validation of novel MKS3/TMEM67 mutations in COACH syndrome
Source: Sci Rep. 2017 Aug 31;7:10222. doi: 10.1038/s41598-017-10652-z (PMC5579020; doi:10.1038/s41598-017-10652-z)
Supplement: Supplementary file 1 — Supplementary data [file 41598_2017_10652_MOESM1_ESM.pdf]

Functional validation of novel *MKS3/TMEM67* mutations in COACH syndrome

So-Hyun Lee, Tai-Seung Nam, Wenting Li, Jung Ha Kim, Woong Yoon, Yoo-Duk Choi, Kun-Hee Kim, Hua Cai, Min Jung Kim, Changsoo Kim, Hyon E. Choy, Nacksung Kim, Kee Oh Chay, Myeong-Kyu Kim and Seok-Yong Choi

Supplementary table 1. Primers and PCR conditions for *TMEM67* sequencing analysis.

Exon	Forward primer (5'→3')	Reverse primer (5'→3')	Amplicon size (bp)	AT (°C)
1	TAGCAACCAAGCAACACGAG	AGGCGGGAGTGTTACTTTTG	393	60
2	TGGGAACTTTATTTTTATTTATCAAGG	TCTGTGGGGAAACATTAAACA	254	59
3	TGGCATTTTGAACTTACATGATT	GCTTGAGCCACTGCAAAAAT	295	59
4	TTGGGTTTTGTTAAATATTTTCTGA	CCTTGGGTTATTGTGAAAACTAAG	300	58
5	CTGAATGAATCTACTCTAATCC	TATGAAAAGGCATAAGCAACTG	236	59
6	ATTCAGTTGCTTATGCCTTTTC	TCTAGCCTGAAATTACTAATGG	209	60
7	TGAGACATTTCCCATTCAACA	CCCTAACACTCCGTTTCAGC	370	59
8	GACTGTTCAGGTTCATGTTAC	AATAACTGCACTGAATTCAGTC	257	59
9	CTCCATTATTAAAACAGTTGTAAC	CAAAATGTAGTTATCCTCTAATG	182	60
10	TTTCAGAGTATTTGACCTGATTTTG	CCTCTTGGCTTTGTCTCAGG	196	59
11	ATCCATGTTCGGGTTTGAGA	GGCAACAAGAGCAAAACTCC	303	60
12	TGCTTGCTAATTTTCAATTGTG	TTGATTTCTGCTCAGGGAAAA	302	59
13	AGAGAACGCCATTGTCAGGT	GTTTTCCCCCTTGGCTATTC	417	59
14	TGTATGTTTAAAGGCCCGGATA	TCAGGATCCATAAGAACTCACC	350	59
15	TGGTAAAACCCAGCTACAAATG	ATTAGGGGAGTCTGGGGAAA	270	59
16	GGCTGACTCCTGTGTCCTTT	TGGTGGGAAGAAAATGAGGT	406	59
17	AGGAGGAAGCAGGTGGTCTT	GAATGGGGAGTTGGGAACTT	394	60
18	GTGGGAGGATCCTGTGAGTC	CGCCAAAGATTTTTGAAAGC	334	59
19	AAGCAGACTTAACGCTGGTAC	CCTTTGCTCTGCAAGGGTAG	213	60
20	CCCTTGCAGAGCAAAGGAG	CATGTAAGTCGCATATAATCAC	233	59
21	TTTCAAGGTGAGTAGGGAGAGG	GCTGCAGTGAACCAAGATCA	468	60
22	CACTGTGCTTTTGGTTGGAA	TGGCCCAAAAATTCAATCAT	300	60
23	TTGTTTTGCAGATGAGTTGCT	CTGGGTGACAGAGGGAGACT	306	59
24	CTGTATTTTCTTTTTGAGGCAG	GACAGAATATATCTGAACTGTAC	221	60
25	GATACCAAGAACATAACACTTTG	GTTTACTGACTTGGTTGACTTG	255	59
26	TTTTCATTTTCTCTCCTGCTGA	GCAAGAGAATGGCATGAACC	389	60
27	CATCCAGATTTTGTCACCAGAA	TCCTTCTTTTGGTGGGGTTA	378	60
28	GATTCAGATACCTGATACATG	GGCCATGATTATACTGAGTC	249	59

AT: annealing temperature used in PCR.

Supplementary table 2. Sequences of primers (5' → 3') used to construct plasmids encoding TMEM67 mutants.

Allele	Sequences
p.Gly132Ala	Forward: GTCACTGTCCCATTGcCCATATTTTAGTG<br>Reverse: CACTAAAATATGGgCAATGGGACAGTGAC
p.Tyr920ThrfsX40	Forward: CAGAGAGGTTTGGTACCCAAC<br>Reverse: AAATAAAATTTTGGCAAGCCAAATCCACAACACAG<br>Forward: GCCAAAATTTTATTTGACTACAAAGACCATGACGGTG<br>Reverse: CAACTCAAATGTCCCACCGGTT

Lower case indicates mutated nucleotides.

Supplementary table 3. Sequences of primers (5' → 3') used for RT-PCR of zebrafish *tmem67*.

Sequences	
Forward:	ATGTGAGTGTCAGGCTGGTT
Reverse:	AACACTTGAGGCAACTGAGG

Supplementary table 4. Sequences of primers (5' → 3') used for quantitative real-time PCR.

Genes	Sequences
ACTIN	Forward: CAGCTAGTGCGAATATCATCT<br>Reverse: TTTCTGTCCCATACCAACC
actin	Forward: TTCACCACCACAGCCGAAAGA<br>Reverse: TACCGCAAGATTCCATACCCA
axin2	Forward: CAAAGCTTTGGCCACTG<br>Reverse: GCGTCTGTAGGACCTGTAGC
gli1	Forward: CAGCACACATCTGAACTC<br>Reverse: GATGACGGTCTGCAGGT
ptch1	Forward: AGTGCTCACGTTCACCACCA<br>Reverse: GTCCCACCGTAGCATGGTCA
ptch2	Forward: GGCACAGAGGATGGAGCACT<br>Reverse: CCTCTGGAGGAATAAG
TMEM67	Forward: TTAAGCTTGCCACCATG<br>Reverse: CGGAGATATCAAAGTACTGG

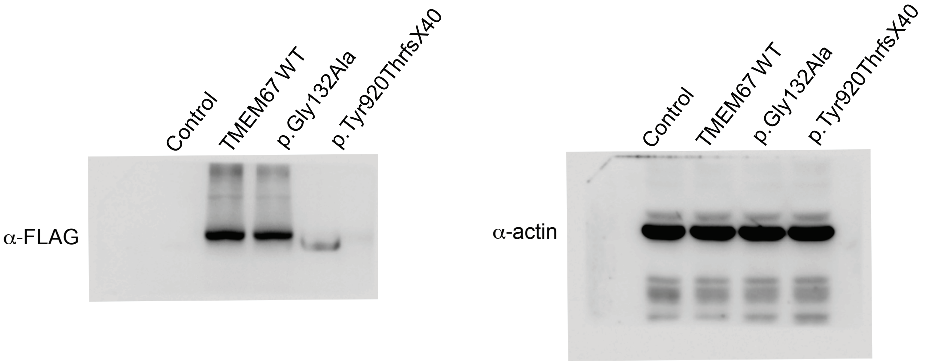

Supplementary Figure S1. Full-length blots that have been cropped in Figure 3A.

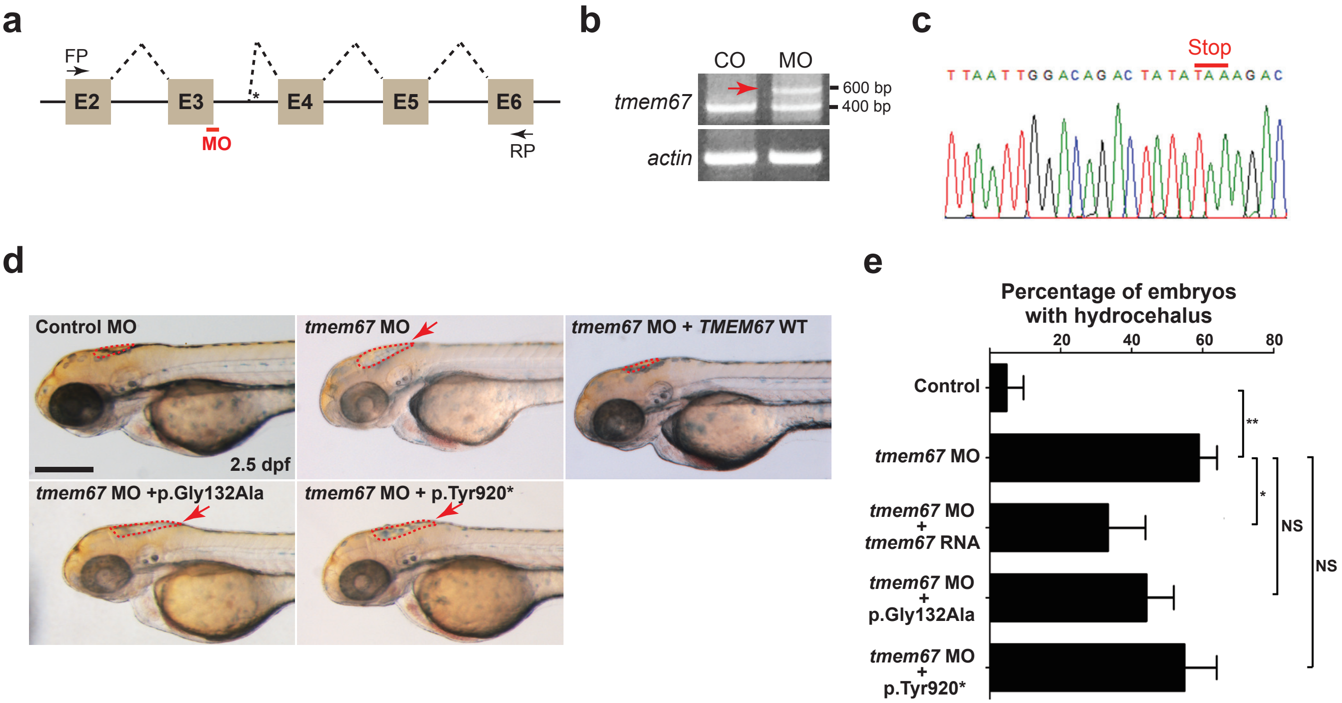

Supplementary Figure S2. TMEM67 mutants show decrease in protein function. (**a**) A schema showing the binding site of *tmem67* splice-blocking morpholino (MO [e3i3]). Dashed lines indicate splicing events. Not drawn to scale. E: exon; FP: forward primer; RP: reverse primer. (**b**) One-cell stage zebrafish embryos were injected with *tmem67* MOs and processed for RT-PCR. Actin was used as a loading control. Arrow indicates a PCR product with deletion of exon3 and retention of a part of intron3. (**c**) An electropherogram of an exon junction area of the PCR product indicated by arrow in (**b**). (**d**) One-cell stage zebrafish embryos were injected with either control MOs or *tmem67* MOs [e3ie] and imaged at 2.5 days post-fertilization (dpf). For a rescue experiment, one-cell stage zebrafish embryos were sequentially injected with *tmem67* MO [e3i3] and RNA encoding the indicated TMEM67 mutant and imaged at 2.5 dpf. Dashed lines mark the hindbrain ventricles. Arrows indicate enlarged ventricles (hydrocephalus). p.Tyr920* represents p.Tyr920ThrfsX40. Scale bar = 100 μm. (**e**) Embryos with hydrocephalus were counted in each group in (**d**). The hindbrain ventricles larger than 5,000 μm^2 were considered to be hydrocephalic. *: $P < 0.05$; ***: $P < 0.001$ by the two-tailed Student's t-test; NS: not significant. Number of larvae used in the analysis of each group is over 40.
